# Solar Spectrum Simulation Algorithms Considering AM0G and AM1.5G

**DOI:** 10.3390/s25051406

**Published:** 2025-02-25

**Authors:** Junjie Yang, Guoyu Zhang, Bin Zhao, Dongpeng Yang, Ke Zhang, Yu Zhang, Jian Zhang, Zhengwei Ren, Jingrui Sun, Lu Wang, Xiaoxu Mo, Taiyang Ren, Dianwu Ren, Zeng Peng, Songzhou Yang, Jiabo Lv

**Affiliations:** 1 School of Optoelectronic Engineering, Changchun University of Science and Technology, Changchun 130022, China; yangjunjie@mails.cust.edu.cn (J.Y.); zb@mails.cust.edu.cn (B.Z.); 2023100377@mails.cust.edu.cn (D.Y.); 2024100408@mails.cust.edu.cn (K.Z.); zhangjian@cust.edu.cn (J.Z.); sunjingrui_cust@163.com (J.S.); wanglu001107@163.com (L.W.); jiuri160@163.com (X.M.); 2022200068@mails.cust.edu.cn (T.R.); 2023100318@mails.cust.edu.cn (D.R.); 18643519878@163.com (Z.P.); szhyang@cust.edu.cn (S.Y.); 2 Jilin Optoelectronic Measurement and Control Instrument Engineering Technology Research Center, Changchun 130022, China; 3 Key Laboratory of Optoelectronic Measurement and Control and Optical Information Transmission Technology, Ministry of Education, Changchun 130022, China; 4 State Key Laboratory of High Power Semiconductor Laser, Changchun University of Science and Technology, Changchun 130022, China; yuzhang@mails.cust.edu.cn; 5 School of Artificial Intelligence, Changchun University of Science and Technology, Changchun 130022, China; renzhengwei@cust.edu.cn; 6 Jilin Province Key Laboratory of Measuring Instrument and Technology, Jilin Institute of Metrology, Changchun 130022, China; lvjiabo003@163.com

**Keywords:** LED solar simulator, multi-objective genetic algorithm, neural network, solar spectrum, spectral simulation

## Abstract

LED solar simulators currently face limitations in their spectral simulation capabilities, especially in terms of accurately incorporating AM0G and AM1.5G solar spectra. To this end, this study introduced a framework for an LED solar spectrum simulation algorithm that considers both AM0G and AM1.5G. This study examined the principle of solar spectrum discretization and reconstruction, established a foundation for analyzing the quality of solar spectrum reconstruction, and developed a non-dominated sorting genetic algorithm II (NSGA-II)-assisted long short-term memory (LSTM)-based solar spectrum simulation strategy. This strategy integrates a multi-objective genetic algorithm to generate training datasets and a neural network for solar spectrum simulation. A dataset generation method using the NSGA-II algorithm was implemented, which leveraged the 6500 K standard blackbody spectral curve, the spectral curve offset coefficients, and the spectral distributions of various narrowband LEDs. An LSTM-based neural network for solar spectrum simulation was developed, with the RMSE serving as the evaluation function. The analysis and selection of 29 narrowband LEDs produced 5000 solar spectrum simulation training datasets. The trained LSTM model achieved spectral matching accuracies within ±10.5% and ±9.3% for AM0G and AM1.5G, respectively, meeting the A+ level simulation standard for solar spectrum reconstruction considering AM0G and AM1.5G. These findings provide a theoretical foundation and technical advancements for high-precision solar spectrum reconstruction, which has practical implications for improving the efficiency and accuracy of solar energy systems, as well as supporting further research on solar spectrum utilization, and is expected to influence the development of more efficient solar simulators.

## 1. Introduction

Solar radiation is integral to aerospace applications [1]; supports advancements in meteorological and environmental sciences [2]; and drives innovations in solar energy conversion, storage, and utilization [3]. However, natural solar radiation is influenced by unpredictable meteorological conditions [4], resulting in intermittency and difficulty in ensuring a stable testing environment [5]. Consequently, solar simulators have emerged as essential research tools, offering controllable, reliable, and repeatable indoor environments for solar radiation illumination and spectral experiments.

The light sources for solar simulators have transitioned from xenon [6] and metal halide light sources [7] to modern LED light sources [8] owing to advancements in LED spectral coverage [9] and energy efficiency [10]. In 2012, Hamadani et al. from the National Institute of Standards and Technology, located in Gaithersburg, MD, USA, proposed a large-area solar simulator using 34 high-power LEDs with a wavelength range of 400 nm–1100 nm to simulate the AM1.5G solar spectrum [11]. In 2019, López-Fraguas et al. of Universidad Carlos III de Madrid achieved a low-cost solar simulator for AM1.5G standards to meet Class AAA performance using 14 different wavelengths of LEDs in Leganés, Spain [12]. In 2021, Tavakoli et al. from Islamic Azad University constructed a spectrally tunable solar simulator in Iran with 19 single-channel high-power LEDs, extending the spectral range to the ultraviolet region [13]. In 2021, Al-Ahmad et al. of University of Newcastle proposed an economical LED solar simulator [14] in Newcastle, Australia, which matched the AM 1.5G solar spectrum within the 350 nm–1100 nm range. Subsequently, Al-Ahmad et al. developed a scalable LED solar simulator meeting the Class A spectral-matching criterion over an area of 320 mm × 200 mm [15]. Thereafter, In 2022, Sun et al. from University of Science and Technology of China designed a high-power narrowband LED-based solar simulator [16] in Hefei, China, achieving Class A AM1.5G solar spectral simulation. In 2023, Du et al. of Beihang University developed a large-area hybrid solar simulator in Beijing, China, which further extended the spectral range [17] with a correlation coefficient of 0.90 between the simulated spectra and reference spectra in the 350 nm–2500 nm range.

Research indicates that with the continuous advancements in LED technology, factors such as the precision of spectral matching to standard solar spectra such as AM0G or AM1.5G, as well as the spectral coverage range, including the ultraviolet and infrared ranges, are no longer the primary factors limiting the spectral performance of LED solar simulators. Significant progress has been made in spectral matching; however, no LED solar simulator has achieved the simultaneous simulation of both AM0G and AM1.5G solar spectra. This limitation arises from inadequacies in existing spectral simulation algorithms. Hence, this study introduces a novel solar spectrum simulation algorithm that is capable of simultaneously considering both AM0G and AM1.5G spectra, offering a significant advancement over existing methods by incorporating a more comprehensive approach to reconstruction and spectral matching. The method of accurately simulating these two key solar spectra using a set of narrowband LEDs is flexible and economical. It also offers the possibility of simulating more kinds of solar spectra in the future and provides the theoretical basis and technical support for further research.

## 2. LED Solar Spectrum Simulation Framework Considering AM0G and AM1.5G

### 2.1. Discrete and Reconstruction Principles

The uniform discrete sampling frequency of the solar spectrum is defined as m, with the sampling wavelength $\lambda_{solari}$ composition matrix represented as $\Delta =\left[\lambda_{solar_{1}},\lambda_{solar_{2}},\cdots ,\lambda_{solar_{m}}\right]$ and the corresponding solar spectral illuminance $\gamma_{i}$ composition matrix denoted as $\omega =\left[\gamma_{1},\gamma_{2},\cdots ,\gamma_{m}\right]$. This formulation enables the solar spectral distribution to be expressed in a matrix form as $X_{solar}=\left[\Delta^{T},\omega^{T}\right]$. Assume the existence of m single-wavelength spectral reconstruction units, $X_{element_{i}}$, denoted as $X_{element_{i}}=\left[\lambda_{solar_{i}},O_{i}\right]$, where $O_{i}$ represents the spectral illuminance at $\lambda_{solar_{i}}$ wavelength, and let the weight coefficient of the spectral reconstruction unit $X_{element_{i}}$ be $l_{element_{i}}$. Hence, the discretized solar spectral reconstruction can be expressed as follows:

(1) $$X_{solar}={\left[\begin{array}{cccc} X_{element_{1}}.L_{element_{1}} & X_{element_{2}}.L_{element_{2}} & \cdots & X_{element_{m}}.L_{element_{m}} \end{array}\right]}^{T}$$

where $L_{element_{i}}$ denotes the matrix of adjustment coefficients, and $L_{element_{i}}=\left[\begin{array}{cc} 1 & 0\\ 0 & l_{element_{i}} \end{array}\right]$. Furthermore, the narrowband LEDs spectral distribution $X_{LED_{i}}$ is sampled based on the wavelength matrix Δ. The spectral illuminance $\beta_{i}$ for a narrowband LED with a peak wavelength of $\lambda_{i}$ at a discrete sampling point is expressed as $\beta_{i}=\left[\begin{array}{cccc} \alpha_{i}\left(\lambda_{solar_{1}}\right) & \alpha_{i}\left(\lambda_{solar_{2}}\right) & \cdots & \alpha_{i}\left(\lambda_{solar_{m}}\right) \end{array}\right]$. The overall narrowband LED spectral illuminance distribution can be written as $X_{LED_{i}}=\left[\Delta^{T},\beta_{i}^{T}\right]$. The reconstructed solar spectral distribution using narrowband LEDs can be expressed as (2) by replacing the spectral reconstruction unit $X_{element_{i}}$ with a narrowband LED featuring a peak wavelength of $\lambda_{i}$ and setting the adjustment coefficient matrix for narrowband LEDs as $L_{LED_{i}}=\left[\begin{array}{cc} \frac{1}{m} & 0\\ 0 & l_{LED_{i}} \end{array}\right]$.

(2) $$X_{solar}=\sum_{i=1}^{m}X_{LED_{i}}L_{LED_{i}}$$

The weight coefficients of narrowband LEDs are precisely adjusted to achieve high-precision matching between the reconstructed spectrum and the target solar spectrum (see (2)).

### 2.2. Basis for Analysis of Reconstruction Quality

Although narrowband LED technology achieves spectral coverage within the wavelength range of 300 nm to 1100 nm [18], the peak wavelength distribution of narrowband LEDs is non-uniform [19]. Therefore, the reconstructed spectral quality of two adjacent narrowband LEDs is analyzed to optimize the selection of narrowband LEDs. The spectral curve $X_{LED_{i}}(\lambda )$ of narrowband LEDs exhibits a normal distribution [20]. The spectral curve is normalized as shown in (3) to enhance the matching speed and generalization ability of the algorithm.

(3) $$X_{LED_{i}}(\mu )=e^{-\frac{{\left(\mu -\mu_{i}\right)}^{2}}{2\eta_{i}^{2}}}$$

where $\mu_{i}$ represents the peak wavelength, and $\eta_{i}$ denotes the standard deviation of the spectral curve. The half-peak full width ω of a narrowband LED is a common measure for characterizing their width (η≈0.425ω). The reconstructed spectra of two adjacent narrowband LEDs $\sum_{j=i}^{i+1}X_{LED_{i}}(\mu )$ can be represented as

(4) $$\sum_{j=i}^{i+1}X_{LED_{i}}(\mu )=e^{-\frac{{\left(\mu -\mu_{1}\right)}^{2}}{0.361{\omega_{1}}^{2}}}+e^{-\frac{{\left(\mu -\mu_{2}\right)}^{2}}{0.361{\omega_{2}}^{2}}}$$

where $\mu_{1}$ and $\mu_{2}$ denote the peak wavelengths, and $\omega_{1}$ and $\omega_{2}$ represent the half-peak full widths of the two LEDs.

The number of narrowband LEDs is reduced while maintaining complete spectral coverage without gaps to ensure high-quality solar spectral reconstruction. Two metrics, spectral intensity uniformity (SD) [21] and spectral purity (SMSR) [22], are employed to assess the reconstruction quality:

(5) $$SD=\sqrt{\frac{1}{N-1}\sum_{\mu =1}^{N}(\sum_{j=i}^{i+1}X_{LED_{i}}(\mu )-\bar{\sum_{j=i}^{i+1}X_{LED_{i}}(\mu )}}$$

(6) $$SMSR=10\cdot \log_{10}\left(\frac{\sum_{j=i}^{i+1}X_{LED_{i},main}\left(\mu \right)}{\sum_{j=i}^{i+1}X_{LED_{i},side}\left(\mu \right)}\right)$$

where N denotes the number of sampled wavelengths, $\bar{\sum_{j=i}^{i+1}X_{LED_{i}}(\mu )}$ represents the average radiance across all sampled wavelengths, $\sum_{j=i}^{i+1}X_{LED_{i},main}(\mu )$ corresponds to the maximum radiance, and $\sum_{j=i}^{i+1}X_{LED_{i},side}(\lambda )$ refers to the subpeak radiance.

**Figure 1:**
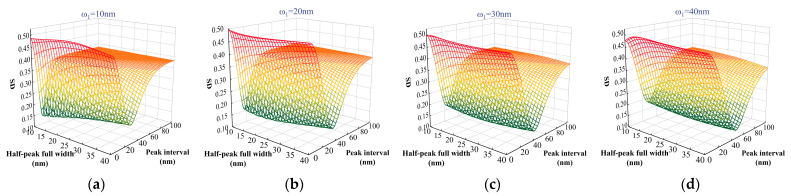

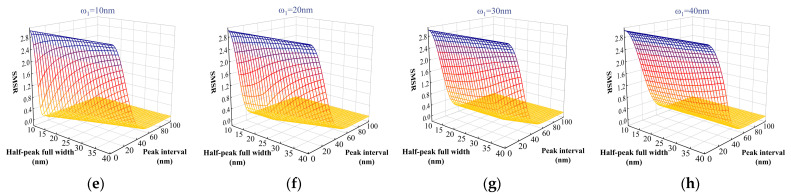
Different colors are used to distinguish between SD and SMSR. SD and SMSR after spectral reconstruction. (**a**–**d**) correspond to $\omega_{1}$ for 10 nm, 20 nm, 30 nm, and 40 nm, respectively, while $\omega_{2}$ represents the SD range from 10 nm to 40 nm, $\sum_{j=i}^{i+1}X_{LED_{i}}(\lambda )$. (**e**–**h**) depict $\omega_{1}$ for 10 nm, 20 nm, 30 nm, and 40 nm, while $\omega_{2}$ indicates SMSR range from 10 nm to 40 nm.

In order to ensure the reconstructed spectral quality, the spectral intensity uniformity and spectral purity of the superposition spectrum from two adjacent narrowband LEDs were analyzed. The peak wavelength and half-peak full width of the next narrowband LED were determined sequentially by considering the relationship between the peak wavelength interval and half-peak full width. The half-peak full width of a narrowband LED is an important parameter for its spectral characterization, which usually ranges from 10 nm to 40 nm. The half-peak full widths of two neighboring LEDs are denoted by $\omega_{1}$ and $\omega_{2}$, respectively, both ranging from 10 nm to 40 nm. To ensure that the reconstructed spectra were continuous and uninterrupted, the range of peak wavelength intervals was set from 0 nm to 100 nm. The spectral reconstruction results are illustrated in Figure 1.

The SD index evaluates the homogeneity of the reconstructed spectrum, with lower values indicating greater homogeneity and smaller intensity differences between wavelengths. The SMSR index quantifies the purity of the reconstructed spectrum, where higher values correspond to fewer spurious peaks beyond the main peaks, resulting in higher spectral purity. Consequently, optimizing the selection of narrowband LED light sources requires a comprehensive consideration of both the SD and SMSR indices to achieve superior solar spectrum reconstruction quality.

### 2.3. Simulation Algorithm Strategy

**Figure 2:**
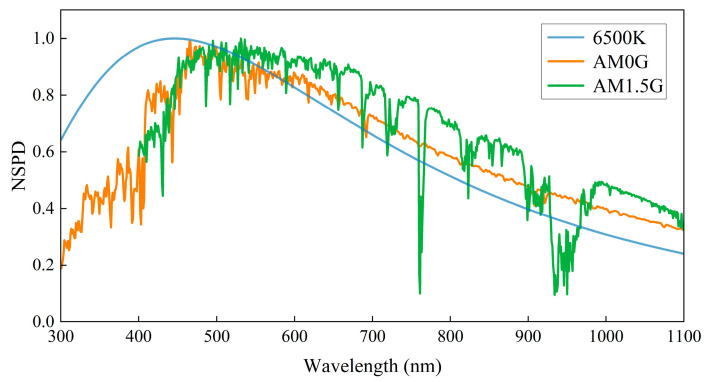
Spectral distributions of 6500 K standard blackbody, AM0G, and AM1.5G solar spectra.

Following standard specifications for spectral irradiance distribution, the wavelength range of the AM0G solar spectrum is defined as 300 nm–1100 nm [23], whereas the AM1.5G solar spectrum covers the range of 400 nm–1100 nm [24]. These solar spectra exhibit complex spectral features that differ significantly from the smooth spectral profile of standard blackbody radiation (e.g., 6500 K) [25]. The standard blackbody radiation curve at 6500 K, along with the normalized spectral power distributions (NSPDs) for AM0G and AM1.5G solar spectra, is depicted in Figure 2.

This study leverages the advantages of multi-LED spectral reconstruction to achieve the efficient and balanced simulation of AM0G and AM1.5G solar spectra while improving system robustness and generality due to the limited availability of publicly accessible training datasets for spectral profile simulation [26]. This study proposes a two-stage solar spectral simulation neural network, incorporating a multi-objective genetic algorithm for the training dataset and an NSGA-II-assisted LSTM simulation algorithm strategy.

The first stage involves employing a multi-objective genetic algorithm, specifically non-dominated sorting genetic algorithm II (NSGA-II), to generate a diverse set of optimal solutions based on various LEDs configurations. This process yields a solar spectrum LSTM simulation training dataset targeting specific spectral curves.

An LSTM neural network architecture is used in the second stage. The LSTM model is trained using the previously generated solar spectrum simulation dataset to enhance the generalization and efficiency of the LSTM-based solar spectrum simulation neural network.

## 3. NSGA-II Assisted LSTM Simulation Algorithm for the Solar Spectrum

### 3.1. NSGA-II-Based Solar Spectrum Simulation Training Method for Generating Datasets

NSGA-II is an efficient multi-objective genetic algorithm designed based on the idea of the genetic algorithm [27]. It maintains the diversity and uniform distribution of superior solutions through non-dominated sorting and crowding degree calculation and has excellent fitness distribution ability. It performs well in maintaining population diversity and avoiding the loss of superior solutions. The algorithm begins with the random generation of an initial population. Then, the objective function values of each individual are calculated. The population is subjected to non-dominated sorting and crowding degree calculation. The next-generation population is selected from the parent and offspring populations to retain excellent individuals and guide the population towards the optimal solution. The selected population is subjected to crossover and mutation operations, where crossover combines the excellent genes of the parent individuals to produce better offspring, and mutation introduces new genetic variations to increase population diversity and prevent the algorithm from falling into a local optimum. The parent and offspring populations are combined to form a temporary population, and the next-generation population is selected by non-dominated sorting and crowding degree calculation. These steps are repeated until the preset number of iterations is reached.

This study developed a multi-objective linear programming model, incorporating the spectral curve of a 6500 K standard blackbody radiation $S_{6500K}(\lambda )$ as an approximation of the solar spectrum, the spectral curve offset coefficient b, and the spectral characteristics of narrowband LEDs $X_{LED_{i}}(\lambda )$ as an input variable. The weight coefficients were determined as outputs, with the root mean square error (RMSE) of the spectral simulation serving as the evaluation function:

(7) $$RMSE=\sqrt{\frac{\sum {\left(b\cdot S_{6500K}(\lambda )-\sum_{i=1}^{m}L_{i}\cdot S_{LED_{i}}(\lambda )\right)}^{2}}{m}},b>1,L_{i}\in \left[0,1\right]$$

The iterative operations of NSGA-II were adjusted by varying the spectral curve offset coefficient b based on the principles of multi-objective linear programming, enabling effective control over the size of the training dataset while maintaining its diversity.

### 3.2. LSTM-Based Neural Network for Solar Spectrum Simulations

**Figure 3:**
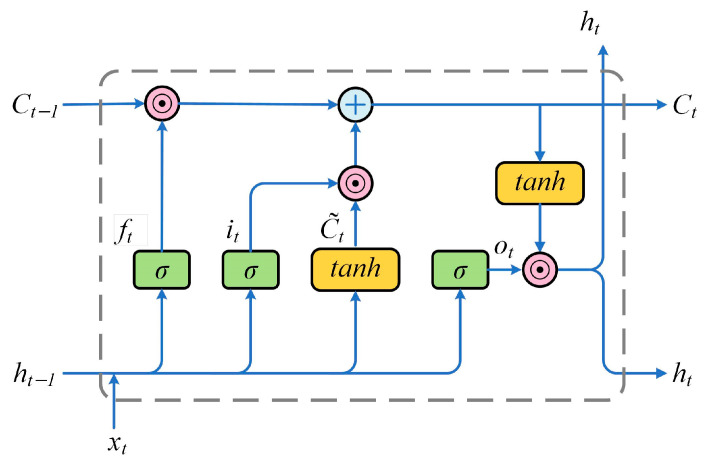
Schematic of structure of LSTM cell as function of applied field.

LSTM, an advanced recurrent neural network, was originally introduced by Hochreiter and Schmidhuber J [28,29]. LSTM incorporates memory storage units and gate structures that address the limitations of traditional recurrent neural networks, such as gradient vanishing and gradient explosion [30,31]. The memory units store state information, while the gate structures regulate information flow selectively through a mechanism of neural layers and element-wise multiplications. The LSTM model employs three types of gates: (1) The forgetting gate determines which information to discard from the memory unit. (2) The input gate identifies and integrates relevant new information into the memory unit. (3) The output gate filters the output information for subsequent processing. This architecture enables LSTM networks to capture long-term dependencies in the input features, enhancing their ability to model complex spectral characteristics. The structure of an LSTM cell, illustrating the interaction between gates and memory states, is shown in Figure 3.

In Figure 3, $x_{t}$ and $h_{t}$ denote the input and output vectors, respectively. Moreover, $f_{t}$, $i_{t}$, and $o_{t}$ represent the activation values of the forgetting gate, input gate, and output gate, respectively; $C_{t}$ and ${\tilde{C}}_{t}$ indicate the cell status and its candidate value, respectively, while the subscript t indicates the time step. The symbols σ and tanh refer to the sigmoid and tanh activation functions, respectively. The weight matrix and bias vector are represented by W and b, respectively, and ⊙ denotes the element-wise multiplication of two matrices.

The updating mechanism of an LSTM cell at each time step t is governed by the following vectorization equations:

Step 1: Compute the activation value of the forgetting gate at the time step t as follows:

(8) $$f_{t}=\sigma (W_{f}\cdot [h_{t-1},x_{t}]+b_{f})$$

Step 2: Determine the new information to be added to the cell state $C_{t}$, including the activation values for the input gate and the candidate state:

(9) $$i_{t}=\sigma (W_{i}\cdot [h_{t-1},x_{t}]+b_{i})$$

(10) $${\tilde{C}}_{t}=\tanh(W_{c}\cdot [h_{t-1},x_{t}]+b_{c})$$

Step 3: Update the cell state $C_{t}$ by combining the previous cell state $C_{t-1}$, the forget gate activation $f_{t}$, the input gate activation $i_{t}$, and the candidate state ${\tilde{C}}_{t}$:

(11) $$C_{t}=f_{t}⊙C_{t-1}+i_{t}⊙{\tilde{C}}_{t}$$

Step 4: Compute the output value $h_{t}$ of the LSTM cell, which determines the flow of information from the cell state $C_{t}$ in the current time step through the activation of the output gate:

(12) $$o_{t}=\sigma (X_{o}\cdot [h_{t-1},x_{t}]+b_{o})$$

(13) $$h_{t}=o_{t}⊙\tanh(C_{t})$$

An LSTM-based solar spectrum simulation neural network was developed based on the classical LSTM neural network architecture by incorporating a fully connected layer, which ensures that the number of nodes matches the number of LEDs types in the training dataset.

The LSTM-based solar spectrum simulation neural network was trained using the training dataset generated by the NSGA-II multi-objective genetic algorithm. This algorithm incorporates multiple LEDs to simulate the solar spectrum accurately, considering both AM0G and AM1.5G standards [23,24].

## 4. Example of Solar Spectrum Simulation

### 4.1. LED Species Selection and Training Dataset Generation

**Figure 4:**
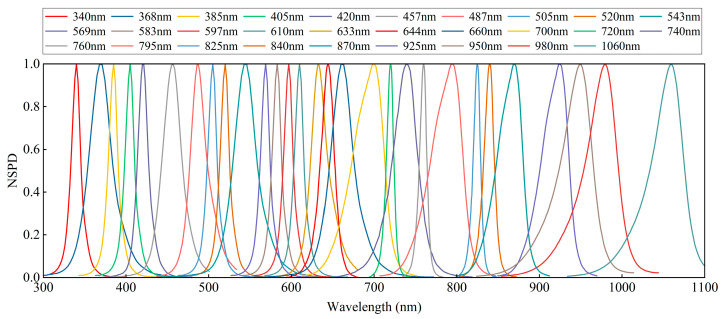
Normalized spectral power distributions of 29 selected narrowband LEDs.

This study selected 29 narrowband LEDs as the base light sources to ensure high-quality solar spectral reconstruction based on (5) and (6) and existing narrowband LED resources. The normalized spectral power distributions (NSPDs) of these LEDs are depicted in Figure 4. The selected LED light source was introduced into an integrating sphere with a radius of 100 mm. The coating material of this integrating sphere is polytetrafluoroethylene, which has an extremely high reflectivity in the UV-VIS-NIR bands (0.25–2.50 μm) and ensures multiple diffuse reflections of the light inside the sphere to achieve a uniform distribution of the spectrum. The spectra at the exit of the integrating sphere were then collected using an AvaSpec-ULS2048XL-EVO fiber optic spectrometer (sourced from Avantes, located in Alphen aan den Rijn, Netherlands).

Considering the spectral range required from 300 nm to 1100 nm, the 6500 K standard blackbody spectral curves and the spectra of 29 selected narrowband LEDs were discretized at 1 nm intervals within this range [32,33]. The spectral curve offset coefficient, b, was randomly assigned values between 1 and 1.5. A comprehensive training dataset was generated for the LSTM-based solar spectral simulation neural network through 5000 iterations.

### 4.2. Neural Network Training and Iterative Simulation Effects

**Figure 5:**
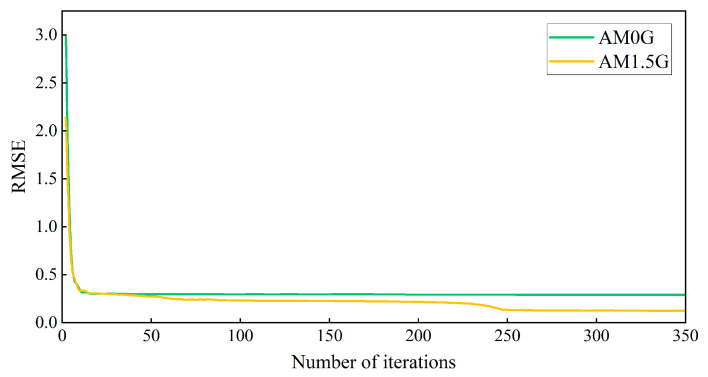
RMSE values as a function of training iterations for simulated AM0G and AM1.5G solar spectra.

Given the selection of 29 narrowband LEDs, the fully connected layer of the LSTM-based solar spectrum simulation neural network was configured with 29 nodes. The AM0G and AM1.5G spectra were inputs to train the LSTM-based solar spectrum simulation neural network using the training dataset from Section 4.1. The RMSEs for the AM0G and AM1.5G solar spectra were recorded as a function of training time, as shown in Figure 5.

The RMSE value for the AM0G model stabilized after 200 iterations during the iterative process, while the RMSE for the AM1.5G model reached a steady state after approximately 250 iterations. Representative solar spectrum simulation curves corresponding to significant changes in the RMSE were selected for analysis to evaluate the simulation performance of the solar spectrum during the iteration process. The iteration times associated with these curves are detailed in Table 1.

**Figure 6:**
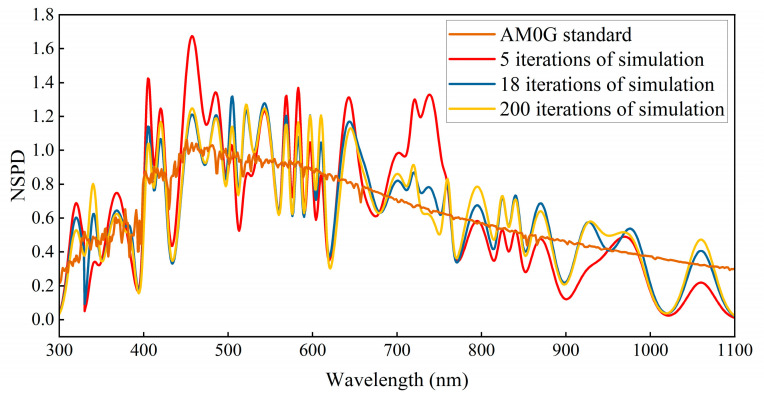
Solar spectrum simulation for varying numbers of AM0G feature iterations.

**Figure 7:**
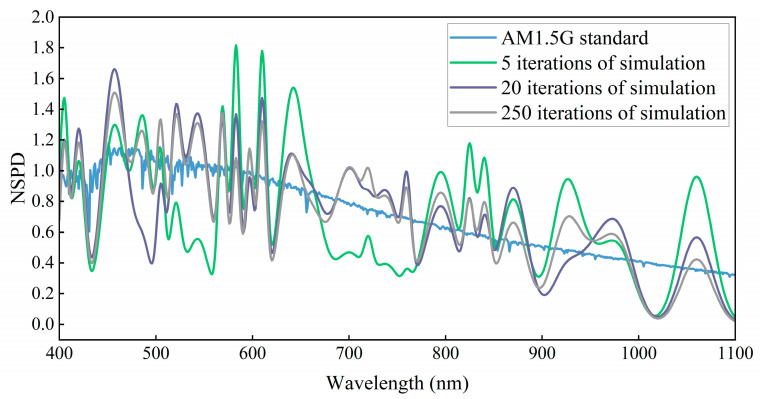
Solar spectrum simulation for varying numbers of AM1.5G feature iterations.

Based on the weight coefficients of the 29 narrowband LEDs during the iteration process, the light source was adjusted to reach an irradiance of one solar constant at the exit of the integrating sphere. The spectra of the LED light sources were acquired using a spectrometer, and the simulated distributions of the AM0G and AM1.5G solar spectra were obtained, as shown in Figure 6 and Figure 7.

### 4.3. Matched Solar Spectrum Simulations

According to the international standard IEC 60904-9:2020, the spectral matching accuracy of solar simulators was evaluated using the “percentage of energy in the interval” metric [23,24]. The specific calculation process is defined in (14) and (15):

(14) $$SPD_{AM0}=\sum_{300nm}^{1100nm}\left|E_{SIM}(\lambda )-E_{AM0}(\lambda )\right|\cdot \Delta \lambda /\sum_{300nm}^{1100nm}\left|E_{AM0}(\lambda )\right|\cdot \Delta \lambda \cdot 100\%$$

(15) $$SPD_{AM1.5}=\sum_{400nm}^{1100nm}\left|E_{SIM}(\lambda )-E_{AM1.5}(\lambda )\right|\cdot \Delta \lambda /\sum_{400nm}^{1100nm}\left|E_{AM1.5}(\lambda )\right|\cdot \Delta \lambda \cdot 100\%$$

**Figure 8:**
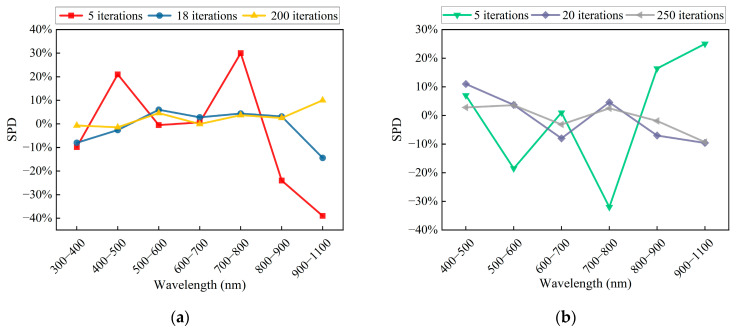
Solar spectral matching performance as a function of feature iterations. (**a**) AM0G and (**b**) AM1.5G.

Figure 6 and Figure 7 provide the necessary data, and the solar spectral matching errors for AM0G and AM1.5G at different feature iteration counts, obtained based on Equations (14) and (15), are shown in Figure 8. Table 2 and Table 3 summarize compliance with A-level criteria (error line ± 25%) or A+-level criteria (error line ± 12.5%) for spectral matching, respectively.

As seen in Table 2 and Table 3, the spectral simulations of both AM0G and AM1.5G have bands that do not meet the A-level standard at the beginning of the iteration (five iterations for both AM0G and AM1.5G). During the mid-iteration stages (18 iterations for AM0G and 20 iterations for AM1.5G), AM0G meets the A+ standard except in the 900–1100 nm range, while AM1.5G meets the A+ standard across all bands but is close to the allowable error limit of the A+ standard in the 400–500 nm and 900–1100 nm ranges. All spectral bands of AM1.5G satisfied the A+-level standard during the later iteration stages (200 iterations for AM0G and 250 iterations for AM1.5G). Specifically, bands within the 300–900 nm range for AM0G achieved the A+-level standard with the spectral simulation deviations constrained within ±4.5%. However, the 900–1100 nm band exhibited a simulation deviation of −10.5%. Similarly, the AM1.5G spectrum maintained deviations better than ±3.6% across all bands except for the 900–1100 nm range, which showed a simulation deviation of −9.3%. The relatively larger deviations in the 900–1100 nm range can be attributed to the limited variety of LEDs types available for this wavelength range among the 29 narrowband LEDs used in this study. The simulation met the A+-level standard despite these limitations. Expanding the variety of narrowband LEDs in the 900–1100 nm range is expected to further enhance matching performance owing to advancements in LED technology [34,35].

## 5. Conclusions and Discussion

This study presents a framework for LED-based solar spectrum simulation incorporating AM0G and AM1.5G spectra. The proposed framework encompasses solar spectrum discretization and reconstruction quality assessment and a solar spectrum simulation algorithm. The principles of solar spectrum discretization and reconstruction were examined, and a discretized reconstruction model was developed. A spectral reconstruction quality assessment method based on SD and SMSR was established using the normal distribution characteristics of narrowband LED spectral distributions as an example. A solar spectrum simulation strategy was also proposed, employing an LSTM neural network and NSGA-II-assisted training dataset generation.

The NSGA-II-based dataset generation method for solar spectrum simulation used the 6500 K standard blackbody spectral curve, the spectral curve offset coefficient, and the spectral distributions of various narrowband LEDs as input variables. The RMSE served as the evaluation function, and a fully connected layer was incorporated into the classical LSTM neural network to form the solar spectrum simulation neural network.

We generated 5000 training datasets for solar spectrum simulation using the 29 narrowband LEDs as the basic light sources. The LSTM-based solar spectrum simulation neural network was trained using this dataset. The results indicated that during AM0G spectral simulation, the RMSE stabilized after 200 iterations. At this stage, the spectral simulation deviation was less than ±4.5% for wavelengths between 300 nm and 900 nm and −10.5% for wavelengths between 900 nm and 1100 nm. All spectral bands achieved the A+-level standard. Similarly, the RMSE for AM1.5G stabilized after 250 iterations, with all spectral bands reaching the A+-level standard. The spectral simulation deviation across all bands was better than ±3.6%, except for the 900–1100 nm range, which exhibited a deviation of −9.3%.

The larger deviations in the 900–1100 nm range for both AM0G and AM1.5G spectra can be attributed to the limited number of narrowband LEDs available in this wavelength range. These deviations are relatively higher than those observed for other spectral bands despite achieving the A+-level standard. Future advancements in LED technology, particularly the development of additional narrowband LEDs within the 900–1100 nm range, are expected to further improve the accuracy of solar spectrum simulations.

Moreover, expanding the simulated wavelength range to a wider range (e.g., from 20 nm to 2000 nm) represents an important research direction in the field of solar spectrum simulation. Multi-junction solar cells are able to utilize sunlight in different wavelength ranges more efficiently due to their unique structural design. This requires a wider wavelength range and higher spectral simulation accuracy in solar spectrum simulation for different types of solar cell technologies.

In future research, we will aim to further optimize solar spectrum simulation techniques to accommodate diverse environmental conditions and meet the needs of specific applications such as agriculture, photovoltaics, and photobiology. We will extend the simulation algorithms to incorporate factors such as geographic location, seasonal variations, and atmospheric effects, enabling the simulator to more accurately replicate the solar spectrum in different regions of the globe and under various environmental conditions.

## Figures and Tables

**Table 1:** Feature iteration counts for AM0G and AM1.5G.

Spectral Type	Number of Feature Iterations
AM0G	5, 18, 200
AM1.5G	5, 20, 250

**Table 2:** Spectral matching performance for varying feature iteration counts of AM0G.

Wave Band/nm	AM0G Standard Spectral Irradiance Distribution/%	5 Iterations		18 Iterations		200 Iterations	
		Matching Error/%	Compliance with Class A/A+ Standards	Matching Error/%	Compliance with Class A/A+ Standards	Matching Error/%	Compliance with Class A/A+ Standards
300–400	9.4%	−9.8%	A+	−8%	A+	−0.7%	A+
400–500	18.5%	21%	A	−2.6%	A+	−1.43%	A+
500–600	18.6%	0.49%	A+	6%	A+	4.5%	A+
600–700	15.8%	0.63%	A+	2.8%	A+	−0.06%	A+
700–800	12.8%	31%	falling short	4.4%	A+	3.7%	A+
800–900	10.2%	24%	A	3.14%	A+	2.5%	A+
900–1100	14.7%	39%	falling short	14.4%	A	−10.5%	A+

**Table 3:** Spectral matching performance for varying feature iteration counts of AM1.5G.

Wave Band/nm	AM1.5G Standard Spectral Irradiance Distribution/%	5 Iterations		20 Iterations		250 Iterations	
		Matching Error/%	Compliance with Class A/A+ Standards	Matching Error/%	Compliance with Class A/A+ Standards	Matching Error/%	Compliance with Class A/A+ Standards
400–500	18.4	7%	A+	11%	A+	2.8%	A+
500–600	19.9	−18.5%	A	3.7%	A+	3.6%	A+
600–700	18.4	1%	A+	−8%	A+	−3.1%	A+
700–800	14.9	−32%	falling short	4.6%	A+	2.5%	A+
800–900	12.5	16.4%	A	−7%	A+	−1.9%	A+
900–1100	15.9	25.2%	falling short	−9.6%	A+	−9.3%	A+

## Data Availability

The data presented in this study are available upon request from the corresponding author.
